# Effect of Korean Red Ginseng on Plasma Ceramide Levels in Postmenopausal Women with Hypercholesterolemia: A Pilot Randomized Controlled Trial

**DOI:** 10.3390/metabo11070417

**Published:** 2021-06-24

**Authors:** Yu-Jin Kwon, Gyung-Min Lee, Kwang-Hyeon Liu, Dong-Hyuk Jung

**Affiliations:** 1 Department of Family Medicine, Yongin Severance Hospital, Yonsei University College of Medicine, Yongin 16995, Korea; digda3@yuhs.ac; 2 BK 21 FOUR Community-Based Intelligent Novel Drug Discovery Education Unit, Research Institute of Pharmaceutical Sciences, College of Pharmacy, Kyungpook National University, Daegu 41566, Korea; lgm00179@naver.com

**Keywords:** Korean red ginseng, ceramides, menopause, cardiovascular diseases, hypercholesterolemia

## Abstract

Cardiovascular disease (CVD) is a crucial cause of death in postmenopausal women. Plasma ceramide concentrations are correlated with the development of atherosclerosis and are significant predictors of CVD. Here, we conducted a 4-week, double-blinded, placebo-controlled clinical pilot study to investigate the effect of Korean red ginseng (KRG) on serum ceramide concentrations in 68 postmenopausal women with hypercholesterolemia. Patients were randomly assigned to two groups: the experimental group (*n* = 36) received KRG and the control (*n* = 32) group received placebo, 2 g each, once daily. Serum ceramides were measured using liquid chromatography–tandem mass spectrometry at baseline and study completion, with changes in serum ceramide levels as the primary end point. We detected significantly greater mean changes in C16 ceramide levels (d18:1/16:0: −6.4 ± 6.3 pmol/mL vs. 14.6 ± 6.8 pmol/mL, respectively, *p* = 0.040; d18:1/22:0: −20.8 ± 24.4 pmol/mL vs. 71.1 ± 26.2 pmol/mL, respectively, *p* = 0.020). Additionally, changes in the median C16 (d18:1/16:0) and C22 (d18:1/22:0) ceramide levels were significantly greater in KRG-group subjects with metabolic syndrome than those without. Therefore, we found that KRG decreases the serum levels of several ceramides in postmenopausal women with hypercholesterolemia, suggesting it may be beneficial for preventing CVD in these individuals.

## 1. Introduction

Cardiovascular disease (CVD) is the leading cause of death for women in the United States [1]. In Korea, more women than men die of CVD, with this disease accounting for 1 in 4.5 deaths in women compared to 1 in 5.8 in men in 2009 [2]. Women also have an increased risk for CVD after menopause and typically develop coronary heart disease several years later compared to men [3]. In the Baltimore Longitudinal Study of Aging, which enrolled participants aged 55 years and older, plasma ceramides concentrations were higher in women than in men [4]. Vozella et al. [5] also found that specific plasma ceramides increased with aging in women, and that the plasma ceramides level were associated with lower estradiol levels in the postmenopausal women.

Plasma ceramides are known to promote atherosclerotic changes through their interactions with lipids, inflammatory cytokines, homocysteines, and matrix metalloproteinases [6]. Laaksonen et al. [7] reported that plasma ceramide species are significant predictors of CVD death beyond traditional lipid markers in patients with stable coronary artery disease and acute coronary syndrome. Ceramide accumulation due to excess fatty acid levels occurs not only in plasma but also in tissues (e.g., skeletal muscle, liver, and adipose tissue), and is closely correlated with insulin resistance [8]. In one study, Haus et al. [9] reported that plasma ceramide levels are increased in patients with type 2 diabetes and correlated with insulin resistance.

Korean red ginseng (KRG) (*Panax ginseng* Meyer, Araliaceae) is commonly used as traditional herbal medicine in Far East Asia. The root of KRG exerts beneficial effects on CVD through its anti-oxidative, anti-inflammatory, and vasomotor regulatory effects [10]. Mass spectrometry-based lipidomic profiling has proven to be a useful method for identifying prognostic markers for CVD [11,12].

However, few studies have investigated the effect of KRG supplementation on lipidomic profiles, particularly ceramides. Moreover, although some reports have suggested that a healthy dietary pattern, such as the Mediterranean diet, can help to improve ceramide levels [13,14], studies investigating the effect of diet or food on plasma ceramides levels are still lacking.

To the best of our knowledge, the current study is the first to prove the effect of KRG on plasma lipid profiles. This study also aimed to measure the effect of KRG treatment on plasma ceramides in subjects with metabolic syndrome, as these individuals tend to have an increased risk for CVD.

## 2. Results

A total of 68 postmenopausal women with hypercholesterolemia participated in this study. Subjects were randomly assigned to either the KRG (*n* = 36) or placebo group (*n* = 32). Table 1 shows the baseline characteristics of the participants in each group. The KRG and placebo groups were similar in terms of age, body mass index (BMI), waist circumference (WC), and blood pressures. The proportions of participants with hypertension or diabetes were also similar in the KRG and placebo groups, and the proportions of physical activity, smoking, or alcohol consumption between the two groups were not significantly different.

Serum concentrations of 12 plasma lipids in participants who received the KRG intervention before and after the 4-week study are shown in Table 2 (KRG group; *n* = 36). We found that only the levels of total plasma ceramides were significantly lowered after 4-week KRG supplementation (1913.9 ± 442.1 pmol/mL vs. 1770.3 ± 410.1 pmol/mL, *p* = 0.025).

Table 3 shows the serum concentrations of six plasma lipids in the KRG and placebo groups before and after the 4-week study. Among the plasma ceramides, mean changes in the levels of C16 ceramide (d18:1/16:0) and C22 ceramide (d18:1/22:0) were significantly greater in the KRG-intervention group than in the placebo group after adjusting for baseline ceramide levels (d18:1/16:0, 6.4 ± 6.3 pmol/mL vs. 14.6 ± 6.8 pmol/mL, respectively, *p* = 0.040; and d18:1/22:0, 20.8 ± 24.4 pmol/mL vs. 71.1 ± 26.2 pmol/mL, respectively, *p* = 0.020).

**Figure 1:**
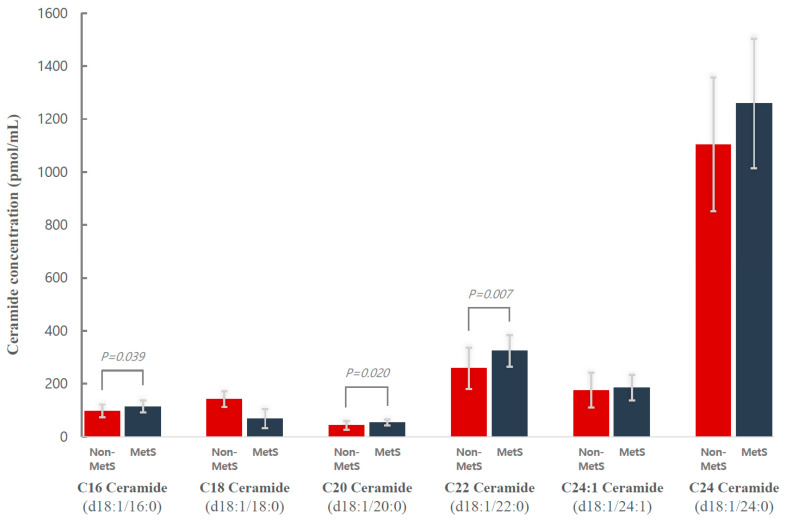
Mean baseline plasma ceramide concentrations in subjects who received Korean red ginseng (KRG) supplementation (*n* = 36) with (*n* = 18) and without metabolic syndrome (*n* = 18). MetS, metabolic syndrome.

Subjects in the KRG-intervention group were then further analyzed, comparing those with metabolic syndrome to those without metabolic syndrome. The mean baseline serum levels of each plasma ceramide species in subjects with and without metabolic syndrome are plotted in Figure 1. The mean levels of C16 ceramide (d18:1/16:0), C20 ceramide (d18:1/20:0), and C22 ceramide (d18:1/22:0) were significantly higher in the metabolic syndrome group than in the non-metabolic syndrome group before intervention (d18:1/16:0, 114.3 ± 22.4 pmol/mL vs. 97.5 ± 24.2 pmol/mL, respectively, *p* = 0.039; d18:1/20:0, 54.0 ± 10.7 pmol/mL vs. 42.4 ± 17.0 pmol/mL, respectively, *p* = 0.020; and d18:1/22:0, 324.8 ± 59.4 pmol/mL vs. 258.8 ± 77.8 pmol/mL, respectively, *p* = 0.007).

Changes in plasma ceramide levels before and after the 4-week KRG intervention in the metabolic syndrome and non-metabolic syndrome groups are shown in Table 4. Among the subjects who received KRG, changes in the median levels of C16 ceramide (d18:1/16:0) and C22 ceramide (d18:1/22:0) were significantly greater in the metabolic syndrome group than in the non-metabolic syndrome group (d18:1/16:0, −10.4 (−31.5, 11.5) vs. 19.2 (−10.4, 47.2), respectively, *p* = 0.019; and d18:1/22:0, −41.1 (−77.4, 15.5) vs. 31.5 (−10.2, 84.4), respectively, *p* = 0.040).

## 3. Discussion

The results of this pilot study provide evidence that KRG supplementation can reduce the levels of two plasma ceramide species—C16 ceramide (d18:1/16:0) and C22 ceramide (d18:1/22:0)—in postmenopausal women with hypercholesterolemia. In addition, we have found that this effect is also detected in the cohort of subjects with metabolic syndrome, with greater reductions in the levels of these ceramides in subjects with metabolic syndrome compared to those without metabolic syndrome.

Several recent studies have identified sphingolipid ceramides as a good prognostic marker for cardiovascular and metabolic disease [7,15], with a number of reports suggesting that ceramide is superior to the traditional marker, low-density lipoprotein cholesterol (LDL-C), for predicting major CVD [7,15,16]. Ceramides comprise a large class of bioactive sphingolipids and play important roles in cell membrane integrity, cellular stress, inflammatory signaling, and apoptosis [6]. Li et al. [17] demonstrated that the accumulation of endogenous ceramides in endothelial cells contributes to the transcytosis of oxidized low-density lipoprotein (LDL) across the endothelial cell barrier and formation of foam cells, thereby promoting atherosclerotic changes in vitro and increasing susceptibility to atherosclerosis in vivo. Consistent with these observations, Schissel et al. [18] analyzed abdominal aortic aneurysm plaque and found that ceramides were elevated in the areas of aggregated LDL compared to plasma LDL.

It was further shown that increased ceramide levels induce production of reactive oxygen species (ROS) by interfering with the mitochondrial electron transport chain, and they can induce apoptosis by altering the permeability of the mitochondrial outer membrane [19]. In addition, ceramides promote atherosclerotic changes by interacting with inflammatory cytokines, such as tumor necrosis factor (TNF)-α. This cytokine increases ceramide formation in vascular endothelial cells by activating the two distinct forms of sphingomyelinase (neutral and acidic sphingomyelinase) and ROS [20]. Ceramides can also mediate the cytotoxicity of TNF-α via ROS generation [21].

Beyond atherosclerosis, ceramides have been associated with type 2 diabetes and insulin resistance [8,9,22]. Lipotoxicity, resulting from the accumulation of bioactive lipids, such as lipoproteins and ceramides, is an important inducer of insulin resistance and β-cell dysfunction [8]. Ceramides also act as antagonists in the insulin signaling pathway by inhibiting activation of serine/threonine kinase Akt/protein kinase B and blocking phosphatidylinositol-3 kinase signaling [23,24].

Several studies have investigated the effect of certain drugs, foods, and diet patterns on plasma ceramides. In one case, lipid-lowering drugs, including statin and fenofibrate, were found to decrease plasma ceramide levels [25,26]. Zhao et al. [27] also demonstrated that 12-week anthocyanin supplementation decreased the plasma ceramide concentrations, especially that of C16 ceramide and C24 ceramide, in a dose-dependent manner in 176 eligible subjects with dyslipidemia. Wang et al. [14] further reported that the Mediterranean diet may have a potential to decrease the harmful effects associated with elevated plasma ceramide levels on CVD risk.

In the current study, we found that 4-week KRG supplementation decreased plasma levels of C16 ceramide (d18:1/16:0) and C22 ceramide (d18:1/22:0) compared to placebo in postmenopausal women with hypercholesterolemia. Notably, these results were also apparent in subjects with metabolic syndrome who received KRG supplementation. Our findings were also consistent with previous studies. Warshauer et al. [22] reported that treatment with pioglitazone, an insulin-sensitizing agent, for 6 months significantly lowered the concentrations of multiple plasma ceramide in 37 subjects with metabolic syndrome, compared to the placebo. Here, the authors suggested that the reduction in ceramide levels may contribute to the mechanism by which pioglitazone improves glucose tolerance and β-cell function. Another study showed that altered ceramide concentrations resulting from high-dose rosuvastatin, a lipid-lowering drug treatment, were inversely associated with the very-low-density lipoprotein (VLDL) apolipoprotein B (apoB)-100 fractional catabolic rate in men with metabolic syndrome, independent of triglycerides and LDL-C [28].

Although the effects of KRG on ceramide metabolism have not been studied, several possible mechanisms could be suggested. A recent review reported that phytonutrients from plant-based diets, especially polyphenols, may decrease the levels of specific ceramides in individuals with metabolic syndrome, type 2 diabetes, and obesity through their medicinal properties, which include anti-inflammatory, anti-oxidative, and anti-diabetic effects [29]. KRG contains ginsenosides, polysaccharides, peptides, alkaloids, polyacetylene, phenols, essential oils, and phytosterols, all of which have demonstrated various pharmacological activities [30]. In particular, the antioxidant activities of KRG have been well studied in in vivo animal and in human clinical studies [31]. As noted above, ceramide increases both ROS and oxidative stress [19,32], whereas inhibition of ROS-generating enzymes or treatment with antioxidants impairs sphingomyelinase activation and ceramide production [29]. Therefore, we speculate that the antioxidant and anti-inflammatory activities of KRG may contribute to the observed reduction in plasma ceramides. Of the various ginsenosides, Rb1, Rg1, Rg3, Rh1, Re, and Rd are well-studied for their molecular mechanisms and medical properties in the treatment of CVDs [10]. Ginsenoside Rb1 prevents ROS toxicity by stimulating nitric oxide (NO) production [33]. Ginsenoside Re acts as an antioxidant and protects cardiomyocytes from oxidant injury induced by both exogenous and endogenous oxidants [34].

**Figure 2:**
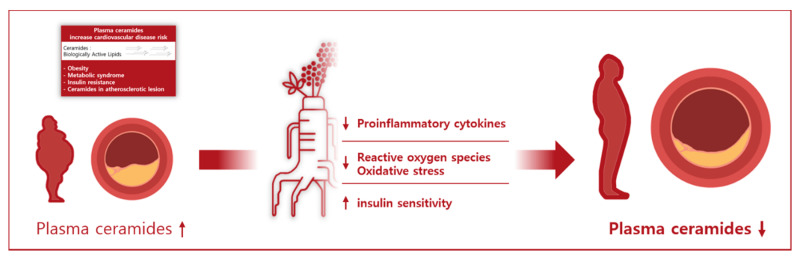
Schematic overview of Korean red ginseng’s effects on plasma ceramides.

Additionally, KRG intervention was found to reduce blood glucose and postprandial glucose [35], and evidence from both in vivo and in vitro studies suggests that KRG regulates blood glucose and enhances glucose uptake by improving β-cell function and up-regulating expression of glucose transporters [35]. Therefore, KRG-mediated improvement in insulin sensitivity could promote beneficial changes in plasma ceramides. Ginsenoside Re alleviates insulin resistance through inhibition of JNK and NF-kappaB activation [36]. Rg1, Rg3, and compound K reduce gluconeogenesis by increasing AMPK expression and decreasing FOXO1 activity [37]. The mechanisms of KRG on plasma ceramides are summarized in Figure 2.

Our study has several limitations. First, it was only conducted for 4 weeks, which is a relatively short period of time. Therefore, it will be essential to identify the time period and schedule for KRG supplementation that are needed to fully observe its effects in human clinical trials. Second, serum ceramide concentrations were influenced by certain drugs and lifestyle modifications, such as dietary changes and exercise, as well as by environmental factors. Here, we only included participants who were not currently taking lipid-lowering drugs; however, we could not control for diet and physical activity. Third, we did not examine the inflammatory markers, such as neutrophil, platelet count, C-reactive protein, or TNF-α, to reveal the mechanism by which KRG affects plasma ceramide. Fourth, we only included Korean postmenopausal women. Therefore, our results cannot be generalized beyond this population. Despite these limitations, this is the first clinical study assessing the effect of KRG on plasma ceramide levels in postmenopausal women with hypercholesterolemia. Future studies with larger sample sizes and more diverse cohorts (e.g., men, other races and ethnicity, and other cardiometabolic diseases) are needed to better understand the effects of KRG on distinct ceramide concentrations. Moreover, further studies to investigate the role of KRG on the whole lipid profiles are needed in both experimental and control groups.

## 4. Materials and Methods

### 4.1. Randomized Trial Design

This is a secondary analysis of a randomized, double-blind, placebo-controlled, 4-week clinical trial evaluating the effect of KRG supplementation on cholesterol metabolites in postmenopausal women with hypercholesterolemia (Clinical Research Information Service (CRIS), KCT0003927) [38]. Detailed information on the study methods and patient recruitment has been published previously [38]. The protocol was approved by the Institutional Review Board (IRB) of Yongin Severance Hospital (Yongin, South Korea), and this study was performed in compliance with the Declaration of Helsinki. Written, informed consent was obtained from all patients prior to participation.

In brief, a total of 84 postmenopausal women who had hypercholesterolemia (serum total cholesterol ≥ 200 mg/dL or serum LDL cholesterol ≥ 130 mg/dL) for at least 1 month and did not take lipid-lowering medications were recruited from October 2018 to May 2019 at Yongin Severance Hospital. Participants were randomly assigned (in a 1:1 ratio) to two groups: one group received four KRG tablets (2 g), and the other received four placebo tablets (2 g) each day. In total, 36 participants in the KRG group and 32 participants in the placebo group completed the study. The KRG dose used in this study was 2 g of KRG tablet/day, containing ginsenoside Rg1 (1.18 mg/g), Rc (3.29 mg/g), Rb1 (8.03 mg/g), Rb2 (2.80 mg/g), Rg3 (2.50 mg/g), Rf (1.47 mg/g), Re (1.29 mg/g), and Rd (1.0 mg/g). KRG tablets were prepared by dehydrating KRG extracts (3 g of KRG extracts per 2 g tablet). Placebo tablets contained corn starch and cellulose with the same flavor.

### 4.2. Study Outcomes

We first investigated possible changes in plasma lipid levels in the KRG group, before and after supplementation, and found that only total plasma ceramide levels were significantly reduced after KRG intervention. We then compared the changes in plasma ceramide levels in the KRG group vs. the placebo group. Lastly, we compared the changes in plasma ceramide levels in subjects from the KRG group with and without metabolic syndrome.

### 4.3. Covariates and Definition of Metabolic Syndrome

Detailed study methods have been described in a previous study [38]. Body weight, WC, SBP, and DBP were measured at each visit. Plasma lipids were measured at baseline and at 4 weeks. Blood samples were obtained after >8 h of fasting. Smoking status, alcohol drinking status, and physical activity were recorded as binary variables. History of hypertension and diabetes were also recorded based on a self-reported questionnaire.

Metabolic syndrome components were defined according to the revised version of the National Cholesterol Education Program Adult Treatment Panel III criteria [39]. In brief, metabolic syndrome was defined as the presence of three or more of the following metabolic syndrome components: (1) waist circumference ≥ 90 cm in men and ≥ 80 cm in women (in accordance with the criteria for the Asian-Pacific population); (2) serum triglycerides ≥ 150 mg/dL or drug treatment for elevated triglycerides; (3) serum HDL-C < 40 mg/dL in men and < 50 mg/dL in women or drug treatment for reduced HDL-C; (4) SBP ≥ 130 mm Hg, DBP ≥ 85 mm Hg, or treatment with antihypertensive medications; and (5) fasting blood glucose ≥ 100 mg/dL or treatment with antidiabetic medications.

### 4.4. Reagents

Lipid standards, including phosphatidylcholine (PC) 25:0 (2:0/13:0), phosphatidylethanolamine (PE) d7-33:1 (d18:0/15:1), lysoPC (LPC) 17:1, lysoPE (LPE) 17:1, sphingomyelin (SM) 30:1 (d18:1/12:0), ceramide (CER) 30:1 (d18:1/12:0), diacylglycerol (DAG) 16:0 (8:0/8:0), triacylglycerol (TAG) 45:0 (15:0/15:0/15:0), cholesteryl ester (CE) 17:0, and d7-acetylcarnitine (AC) 2:0 were obtained from Avanti Polar Lipids (Alabaster, AL, USA), Sigma-Aldrich (St. Louis, MO, USA), or Cayman Chemicals (Ann Arbor, MI, USA). Ammonium acetate, butylated hydroxytoluene, chloroform, and methyl-tert butyl ether (MTBE) were purchased from Sigma Chemical Co. (St. Louis, MO, USA).

### 4.5. Lipid Extraction

Plasma lipids were extracted using the Matyash method, with slight modifications [40,41]. In brief, human plasma (10 μL) was aliquoted into microcentrifuge tubes containing ice-cold 75% methanol (400 μL) with 0.1% butylated hydroxytoluene. MTBE (1 mL) was then added, and the mixture was shaken for 1 h at room temperature. For phase separation, 250 μL water was added, and tubes were centrifuged. Aliquots of the upper phase (110 μL) and lower phase (55 μL) were transferred to new tubes, dried under a nitrogen stream, and reconstituted in 100 μL chloroform/methanol (1:9, *v*/*v*), containing the internal lipid standard (IS) mixture (40–400 ng/mL).

### 4.6. Liquid Chromatography–Tandem Mass Spectrometry (LC-MS/MS)

Semi-quantitative lipid profiling was performed using previously developed methods, with slight modifications [41]. Briefly, all lipids and the IS were separated on a Kinetex C18 column and analyzed using an LC-MS/MS system (LCMS 8060; Shimadzu, Kyoto, Japan). The mobile phase A consisted of a water/methanol mixture (1:9, *v*/*v*), with 10 mM ammonium acetate, and the mobile phase B consisted of an isopropanol/methanol mixture (5:5, *v*/*v*), with 10 mM ammonium acetate. The gradient elution was as follows: 0 min (30% B), 0–15 min (95% B), 15–20 min (95% B), and 20–25 min (30% B). The flow rate was 0.2 mL/min. Quantitation was performed in the selected reaction monitoring (SRM) of the [M + H]^+^ (or [M + NH_4_^+^]) ion and the related product ion for each lipid and IS.

To determine the concentration of each target lipid species, the calculated ratio of target analyte to IS was multiplied by the concentration of the IS [41,42]. An IS for each lipid class was selected for single-point calibrations of each target lipid species. The SRM transitions and collision energies determined for each lipid are listed in Supplemental Table S1.

### 4.7. Statistical Analysis

Data are presented as the means ± standard deviations. Differences within groups after intervention was determined using paired *t*-tests. Statistical significance for differences in baseline characteristics between the KRG group and placebo group was determined using independent *t*-tests for continuous variables and chi-squared tests for categorical variables. Statistical significance for comparison of changes in plasma ceramide levels between two groups was determined using one-way analysis of variance (ANOVA), after adjusting for the baseline levels of each plasma ceramide.

In the subgroup analysis comparing the changes in ceramide levels in subjects with and without metabolic syndrome, the data are presented as medians and interquartile ranges (IQR). Statistical significance for comparison of within-group changes after intervention was calculated using the Wilcoxon signed-rank test. Significance tests were two-sided, with an alpha value of 0.05. All statistical analyses were performed with SPSS software v.25.0 (IBM Corp., Armonk, NY, USA).

## 5. Conclusions

The present study reported significant reductions in plasma C16 ceramide (d18:1/16:0) and C22 ceramide (d18:1/22:0) levels in subjects who received KRG supplementation relative to the placebo group. Moreover, the changes in plasma C16 ceramide (d18:1/16:0) and C22 ceramide (d18:1/22:0) levels were significantly greater in subjects with metabolic syndrome than in those without metabolic syndrome within the KRG group. These results indicate that KRG intake may help to prevent cardiovascular diseases by modulating the circulating levels of ceramides.

## Figures and Tables

**Table 1:** Comparison of the baseline characteristics of study participants in the KRG vs. placebo groups.

	Ginseng *(n* = 36)	Placebo (*n* = 32)	*p*-Value ^1^
Age, years	55.9 ± 5.9	58.1 ± 4.7	0.093
Body mass index (kg/m^2^)	24.3 ± 3.2	24.5 ± 3.7	0.741
Waist circumference (cm)	82.5 ± 8.7	82.6 ± 10.2	0.950
SBP (mmHg)	119.8 ± 13.5	116.8 ± 16.5	0.409
DBP (mmHg)	76.7 ± 9.7	72.2 ± 9.3	0.065
Fasting glucose (mg/dL)	108.4 ± 18.5	103.0 ± 10.2	0.145
Triglycerides (mg/dL)	124.9 ± 60.4	147.7 ± 82.5	0.197
HDL cholesterol (mg/dL)	64.5 ± 14.2	58.6 ± 14.7	0.098
WBC (×10^3^ L)	5.7 ± 1.4	5.8 ± 1.6	0.786
Hypertension	5 (13.9)	5 (15.6)	0.572
Diabetes	2 (5.6)	1 (3.1)	0.535
Physical activity, *n* (%)	15 (41.7)	10 (31.3)	0.374
Smoking, *n* (%)	2 (5.6)	1 (3.1)	0.534
Alcohol consumption, *n* (%)	9 (25.0)	10 (13.2)	0.567
Ceramides (pmol/mL)			
C16 ceramide (d18:1/16:0)	105.9 ± 24.5	154.5 ± 50.9	<0.001
C18 ceramide (d18:1/18:0)	105.6 ± 223.5	64.3 ± 25.1	0.303
C20 ceramide (d18:1/20:0)	48.2 ± 15.2	63.2 ± 22.3	0.002
C22 ceramide (d18:1/22:0)	291.8 ± 76.0	510.7 ± 204.9	<0.001
C24 ceramide (d18:1/24:0)	1181.6 ± 257.3	1594.5 ± 580.9	<0.001
C24:1 ceramide (d18:1/24:1)	180.8 ± 57.0	658.8 ± 225.6	<0.001

^1^*p*-values were calculated using the independent two-sample *t*-test for continuous values and the chi-square test for categorical values. DBP, diastolic blood pressure; HDL, high-density lipoprotein; SBP, systolic blood pressure.

**Table 2:** Plasma lipid profiles before and after intervention in the Korean red ginseng (KRG) group (*n* = 36). Mean serum concentrations for each lipid and associated *p*-values are shown.

Lipids	Before	After	*p*-Value ^1^
Neutral lipids			
Monoacylglycerol (nmol/mL)	50.7 ± 17.6	50.4 ± 12.0	0.931
Diacylglycerol (nmol/mL)	4.1 ± 1.5	4.1 ± 1.6	0.816
Triacylglycerol (nmol/mL)	42,250.9 ± 19,080.588	43,081.1 ± 19,820.933	0.766
Acylcarnitine (nmol/mL)	17.4 ± 9.1	18.1 ± 5.6	0.648
Phospholipids			
Phosphatidylcholine (μmol/mL)	2033.8 ± 435.8	2124.3 ± 634.6	0.309
Plasmenyl phosphatidylcholine (μmol/mL)	93.2 ± 39.8	95.4 ± 25.6	0.730
Lysophosphatidylcholine (nmol/mL)	109.0 ± 25.4	116.8 ± 23.0	0.088
Phosphatidylethanolamine (nmol/mL)	29.8 ± 11.1	29.6 ± 11.7	0.917
Plasmenyl phosphatidylethanolamine (nmol/mL)	40.9 ± 16.7	40.4 ± 13.2	0.832
Lysophosphatidylethanolamine (nmol/mL)	5.6 ± 1.6	6.2 ± 1.9	0.066
Sphingolipids			
Sphingomyelin (μmol/mL)	690.2 ± 143.0	748.5 ± 221.1	0.080
Ceramide (μmol/mL)	1913.9 ± 442.1	1770.3 ± 410.1	0.025

^1^*p*-values were calculated using the paired *t*-test.

**Table 3:** Comparison of changes in serum ceramide levels in the KRG and placebo groups before and after the 4-week intervention.

	Ginseng (*n* = 36)			Placebo (*n* = 32)			
	Pre- Intervention	Post- Intervention	ΔChanges	Pre- Intervention	Post- Intervention	ΔChanges	*p* ^1^
**Ceramides**							
Ceramide (d18:1/16:0)	111.9 ± 34.1	105.9 ± 24.5	−6.4 ± 6.3	154.5 ± 50.9	155.2 ± 43.9	14.6 ± 6.8	0.040
Ceramide (d18:1/18:0)	105.6 ± 2223.5	48.4 ± 18.8	−66.0 ± 29.7	64.3 ± 25.1	58.3 ± 24.7	3.9 ± 31.8	0.142
Ceramide (d18:1/20:0)	48.2 ± 15.2	46.1 ± 15.5	−5.8 ± 3.3	63.2 ± 22.3	59.8 ± 21.8	0.7 ± 3.5	0.214
Ceramide (d18:1/22:0)	291.8 ± 76.0	289.9 ± 80.0	−20.8 ± 24.4	510.7 ± 204.9	560.6 ± 214.3	71.1 ± 26.2	0.020
Ceramide (d18:1/24:0)	1181.6 ± 257.3	1109.4 ± 295.9	−94.3 ± 77.1	1594.5 ± 580.9	1711.0 ± 682.9	141.3 ± 82.6	0.058
Ceramide (d18:1/24:1)	180.8 ± 57.0	164.7 ± 55.8	−40.3 ± 27.7	658.8 ± 225.6	651.6 ± 232.6	20.1 ± 29.7	0.173

^1^*p*-values comparing changes in ceramide levels (ΔChange) in the KRG and placebo groups were calculated using one-way analysis of variance (ANOVA), after adjusting for baseline levels of each ceramide.

**Table 4:** Comparison of changes in serum ceramide levels before and after the 4-week intervention in subjects from the KRG group (*n* = 36) with and without metabolic syndrome ^1^.

	Metabolic Syndrome Group (*n* = 18)			Non-Metabolic Syndrome Group (*n* = 18)			
Parameters	Pre- Intervention	Post- Intervention	ΔChanges	Pre- Intervention	Post- Intervention	ΔChanges	*p* ^2^
Ceramide (d18:1/16:0)	115.1 (90.5, 131.4)	103.6 (88.4, 121.3)	−10.4 (−31.5, 11.5)	95.6 (78.7, 114.8)	119.8 (78.2, 150.0)	19.2 (−10.4, 47.2)	0.019
Ceramide (d18:1/18:0)	65.9 (51.9, 80.1)	46.9 (40.0, 65.8)	−11.7 (−25.7, 1.2)	51.1 (38.6, 58.7)	37.7 (29.4, 50.3)	−9.1 (−30.3, 6.6)	0.999
Ceramide (d18:1/20:0)	53.1 (48.0, 59.4)	47.5 (42.2, 60.0)	0.9 (−16.0, 5.8)	42.7 (29.2, 54.6)	39.7 (29.9, 52.1)	−4.7 (−11.0, 18.3)	0.728
Ceramide (d18:1/22:0)	320.0 (286.0, 357.3)	282.9 (255.6, 365.8)	−41.1 (−77.4, 15.5)	243.0 (205.4, 310.3)	253.0 (228.8, 349.5)	31.5 (−10.2, 84.4)	0.040
Ceramide (d18:1/24:0)	1246.9 (1138.0, 1381.4)	1053.8 (879.2, 1304.3)	−198.6 (−399.0, 38.9)	1102.6 (973.1, 1250.3)	1086.6 (858.1, 1401.5)	−24.5 (−213.9, 236.1)	0.066
Ceramide (d18:1/24:1)	180.2 (157.6, 213.4)	160.2 (117.0, 212.5)	−25.4 (−64.2, 13.7)	171.6 (129.5, 208.0)	161.5 (131.5, 186.7)	−16.6 (−56.7, 36.0)	0.359

^1^ Data are expressed as the median (interquartile range). ^2^ *p*-values comparing the changes in ceramide levels (ΔChanges) in the KRG and placebo groups were calculated using the Wilcoxon rank-sum test.

## Data Availability

The data presented in this study are available on request from the corresponding author. The data are not publicly available since the data needs further use.

## Supplementary Materials

The following are available online at https://www.mdpi.com/article/10.3390/metabo11070417/s1, Table S1: Selected reaction monitoring (SRM) transitions and collision energies for each lipid, Figure S1: HPLC graph of KRG tablets used in this study.
