# Supplementary material for: Effect of Korean Red Ginseng on Plasma Ceramide Levels in Postmenopausal Women with Hypercholesterolemia: A Pilot Randomized Controlled Trial
Source: Metabolites. 2021 Jun 24;11(7):417. doi: 10.3390/metabo11070417 (PMC8307748; doi:10.3390/metabo11070417)
Supplement: Supplementary file 1 [file metabolites-11-00417-s001.zip › Supplemetary Table S1.pdf]

Supplementary Table S1. Selected reaction monitoring (SRM) transitions and collision energies for each lipid.

No.	Class	Species	Adduct ion	Q1 (*m/z*)	Q3 (*m/z*)	CE (eV)	Retention time (min)
1	PC	25:0 (12:0/13:0)	[M+H$^+$]	636.5	184.1	30	2.72
2	LPC	17:1	[M+H$^+$]	508.4	184.1	25	1.58
3	PE	d_7-33:1 (18:0/15:1)	[M+H$^+$]	711.6	570.5	20	5
4	LPE	17:1	[M+H$^+$]	466.4	325.3	25	1.57
5	CE	17:0	[M+H$^+$]	656.6	369.3	18	16.7
6	SM	30:1 (18:1/12:0)	[M+H$^+$]	647.6	184.1	32	2.92
7	DG	16:0 (8:0/8:0)	[M+NH$_4^+$]	362.3	327.3	22	1.67
8	TG	45:0 (15:0/15:0/15:0)	[M+NH$_4^+$]	782.6	523.5	30	14.7
9	CER	30:1 (18:1/12:0)	[M+H$^+$]	482.5	264.3	25	3.33
10	AC	d_7-2:0	[M+H$^+$]	207.2	85.1	27	1.1
11	Gal-CER	18:1/12:0	[M+H$^+$]	644.5	264.3	35	2.75
12	SUL	d_3-18:1/18:0	[M+H$^+$]	811.6	264.3	45	3.57
13		30:2		758.5	184.1	30	3.79
14		30:1		760.5	184.1	30	4.41
15		30:0		762.5	184.1	30	4.8
16		32:2		780.5	184.1	30	4.11
17		32:1		782.5	184.1	30	4.78
18		32:0		784.5	184.1	30	5.54
19		34:4		786.5	184.1	30	4.03
20		34:3		788.5	184.1	30	4.46
21		34:2		806.5	184.1	30	5.04
22		34:1		808.5	184.1	30	5.73
23		34:0		810.5	184.1	30	6.61
24	PC	36:5	[M+H$^+$]	812.5	184.1	30	4.41
25		36:4		814.5	184.1	30	4.94
26		36:3		816.5	184.1	30	5.33
27		36:2		818.5	184.1	30	6.07
28		36:1		834.5	184.1	30	6.82
29		36:0		836.5	184.1	30	7.67
30		38:7		838.5	184.1	30	4.06
31		38:6		840.5	184.1	30	4.81
32		38:5		842.5	184.1	30	5.23
33		38:4		844.5	184.1	30	5.99
34		38:3		742.6	184.1	30	6.44
35		38:2		744.6	184.1	30	7.12

36		38:1		746.6	184.1	30	5.02
37		40:8		766.6	184.1	30	4.34
38		40:7		768.6	184.1	30	4.99
39		40:6		770.6	184.1	30	5.84
40		40:5		772.6	184.1	30	6.15
41		40:4		774.6	184.1	30	6.82
42	Plasmenyl PC	30:2	[M+H$^+$]	686.6	184.1	30	4.43
43		30:1		688.6	184.1	30	3.98
44		30:0		690.6	184.1	30	3.98
45		32:1		716.6	184.1	30	5.25
46		32:0		718.6	184.1	30	6.08
47		34:3		740.6	184.1	30	4.85
48		34:2		742.6	184.1	30	5.55
49		34:1		744.6	184.1	30	6.29
50		34:0		746.6	184.1	30	6.45
51		36:5		764.6	184.1	30	4.83
52		36:4		766.6	184.1	30	5.41
53		36:2		770.6	184.1	30	5.93
54		36:1		772.6	184.1	30	6.8
55		36:0		774.6	184.1	30	7.05
56		38:6		790.6	184.1	30	5.26
57		38:5		792.6	184.1	30	5.52
58		38:4		794.6	184.1	30	5.78
59		38:1		800.6	184.1	30	7.31
60		40:7		816.6	184.1	30	5.02
61		40:6		818.6	184.1	30	5.67
62		40:5		820.6	184.1	30	6
63		40:4		822.6	184.1	30	6.89
64		40:3		824.6	184.1	30	7.88
65	LPC	16:1	[M+H$^+$]	494.3	184.1	25	1.52
66		16:0		496.3	184.1	25	1.64
67		18:3		518.3	184.1	25	1.46
68		18:2		520.3	184.1	25	1.58
69		18:1		522.3	184.1	25	1.7
70		18:0		524.3	184.1	25	1.93
71		20:5		542.3	184.1	25	1.46
72		20:4		544.3	184.1	25	1.52
73		20:3		546.3	184.1	25	1.64
74		22:6		568.3	184.1	25	1.52
75	PE	34:3	[M+H$^+$]	714.6	573.5	20	4.33
76		34:2		716.6	575.5	20	4.87
77		34:1		718.6	577.5	20	5.53
78		36:5		738.6	597.5	20	4.27
79		36:4		740.6	599.5	20	4.78

80		36:3		742.6	601.5	20	5.09
81		36:2		744.6	603.5	20	5.89
82		36:1		746.6	605.5	20	6.58
83		38:6		764.6	623.5	20	4.69
84		38:5		766.6	625.5	20	5.16
85		38:4		768.6	627.5	20	5.8
86		38:3		770.6	629.5	20	6.22
87		40:7		790.6	649.5	20	4.85
88		40:6		792.6	651.5	20	5.67
89		40:5		794.6	653.5	20	5.98
90		40:4		796.6	655.5	20	6.61
91	LPE	16:0	$[M+H^+]$	454.3	313.2	25	1.63
92		18:2		478.3	337.2	25	1.51
93		18:1		480.3	339.2	25	1.67
94		18:0		482.3	341.2	25	1.88
95		22:6		526.3	385.2	25	1.49
96	Plasmenyl PE	34:3 (16:0/18:3)	$[M+H^+]$	698.5	335.2	25	4.79
97		34:2 (16:0/18:2)		700.5	337.2	25	5.36
98		34:1 (16:0/18:1)		702.5	339.2	25	6.1
99		36:5 (16:0/20:5)		722.6	359.2	25	4.69
100		36:4 (16:0/20:4)		724.6	361.2	25	5.28
101		36:3 (16:0/20:3)		726.6	363.2	25	5.73
102		38:6 (16:0/22:6)		748.6	385.2	25	5.13
103		38:5 (16:0/22:5)		750.6	387.2	25	5.46
104		38:4 (16:0/22:4)		752.6	389.2	25	6.1
105		36:3 (18:0/18:3)		726.6	335.2	25	5.8
106		36:2 (18:0/18:2)		728.6	337.2	25	6.43
107		36:1 (18:0/18:1)		730.6	339.2	25	7.2
108		38:5 (18:0/20:5)		750.6	359.2	25	5.71
109		38:4 (18:0/20:4)		752.6	361.2	25	6.32
110		38:3 (18:0/20:3)		754.6	363.2	25	6.78
111		40:6 (18:0/22:6)		776.6	385.2	25	6.17
112		34:1 (18:1/16:0)		702.5	313.2	25	6.09
113		36:3 (18:1/18:2)		726.6	337.2	25	5.56
114		36:2 (18:1/18:1)		728.6	339.2	25	6.3
115		38:6 (18:1/20:5)		748.6	359.2	25	4.89
116		38:5 (18:1/20:4)		750.6	361.2	25	5.49
117		38:4 (18:1/20:3)		752.6	363.2	25	5.94
118		40:7 (18:1/22:6)		774.6	385.2	25	5.34
119		40:6 (18:1/22:5)		776.6	387.2	25	5.65
120		40:5 (18:1/22:4)		778.6	389.2	25	6.28
121	CER	34:2	$[M+H^+]$	536.5	264.3	25	4.61

122		34:1		538.5	264.3	25	5.07
123		36:1		566.5	264.3	25	6.13
124		38:1		594.5	264.3	25	7.28
125		40:2		620.6	264.3	25	7.85
126		40:1		622.6	264.3	25	8.46
127		42:2		648.6	264.3	25	9.05
128		42:1		650.6	264.3	25	9.66
129		34:2		701.6	184.1	32	3.78
130		34:1		703.6	184.1	32	4.42
131		34:0		705.6	184.1	32	4.84
132		36:3		727.6	184.1	32	4.01
133		36:2		729.6	184.1	32	4.64
134		36:1		731.6	184.1	32	5.39
135	SM	38:3	[M+H$^+$]	755.6	184.1	32	4.01
136		38:2		757.6	184.1	32	4.46
137		38:1		759.6	184.1	32	5.05
138		40:3		783.6	184.1	32	4.92
139		40:2		785.6	184.1	32	5.33
140		40:1		787.6	184.1	32	6.08
141		40:0		789.6	184.1	32	6.84
142		34:2		860.6	264.3	42	3.52
143		34:1		862.6	264.3	42	3.84
144	Lac-CER	40:1	[M+H$^+$]	946.6	264.3	42	5.01
145		42:2		972.6	264.3	42	6.79
146		42:1		974.6	264.3	42	7.77
147		34:2		698.5	264.3	42	3.94
148		34:1		700.5	264.3	42	4.18
149		36:2		726.5	264.3	42	4.43
150		36:1		728.5	264.3	42	5.12
151	Gal-CER	38:1	[M+H$^+$]	756.5	264.3	42	6.13
152		40:2		782.5	264.3	42	6.91
153		40:1		784.5	264.3	42	7.21
154		42:2		810.6	264.3	42	8.01
155		42:1		812.5	264.3	42	8.34
156	SUL	34:1		780.5	264.3	45	7.8
157		36:1		808.5	264.3	45	7.02
158		34:2		794.5	264.3	45	8.36
159		40:2	[M+H$^+$]	878.5	264.3	45	15.03
160	HSUL	40:1		880.5	264.3	45	15.69
161		42:2		906.5	264.3	45	15.85
162		42:1		908.5	264.3	45	16.51
163		16:1		640.6	369.3	18	15.31
164	CE	16:0	[M+H$^+$]	642.6	369.3	18	16.2
165		18:3		664.6	369.3	18	14.83

166		18:2		666.6	369.3	18	15.57
167		18:1		668.6	369.3	18	16.35
168		18:0		670.6	369.3	18	17.18
169		20:5		688.6	369.3	18	14.54
170		20:4		690.6	369.3	18	15.29
171		20:3		692.6	369.3	18	15.85
172		20:2		694.6	369.3	18	16.56
173		22:6		714.6	369.3	18	14.98
174		22:5		716.6	369.3	18	15.44
175		22:4		718.6	369.3	18	16.12
176	MG	16:1	$[M+NH_4^+]$	346.3	311.3	18	1.11
177		16:0		348.3	313.3	18	1.95
178		18:3		370.3	335.3	18	1.44
179		18:1		374.3	339.3	18	2.01
180		18:0		376.3	341.3	18	2.32
181	DG	32:2	$[M+NH_4^+]$	582.5	547.5	22	5.72
182		32:1		584.5	549.5	22	6.53
183		32:0		586.5	551.5	22	7.44
184		34:3		608.5	573.5	22	5.99
185		34:2		610.5	575.5	22	6.85
186		34:1		612.5	577.5	22	7.71
187		36:4		634.5	599.5	22	6.34
188		36:3		636.5	601.5	22	7.12
189	TG	46:2 (16:1/14:0/16:1)	$[M+NH_4^+]$	792.7	521.5	30	13.58
190		46:1 (14:0/16:1/16:0)		794.7	549.5	30	14.36
191		46:0 (16:0/14:0/16:0)		796.7	523.3	30	15.16
192		48:3 (16:1/18:2/14:0)		818.7	547.4	30	13.81
193		48:2 (16:1/14:0/18:1)		820.7	549.4	30	14.53
194		48:1 (16:0/14:0/18:1)		822.7	549.4	30	15.32
195		48:0 (16:0/16:0/16:0)		824.7	551.3	30	16.04
196		50:4 (16:1/18:2/16:1)		844.7	573.4	30	14.05
197		50:3 (16:1/18:1/16:1)		846.7	575.4	30	14.77
198		50:2 (18:1/16:1/16:0)		848.7	549.4	30	15.46
199		50:1 (16:0/18:1/16:0)		850.8	577.5	30	16.18
200		50:0 (18:0/14:0/18:0)		852.8	551.3	30	16.85

201		52:4 (18:2/16:1/18:1)		872.7	575.4	30	15.02
202		52:3 (18:1/16:1/18:1)		874.8	575.4	30	15.69
203		52:2 (18:1/16:0/18:1)		876.8	577.4	30	16.31
204		52:1 (16:0/18:1/18:0)		878.8	605.5	30	16.97
205		54:7 (20:4/16:1/18:2)		894.7	573.5	30	14.2
206		54:6 (20:4/16:0/18:2)		896.7	575.5	30	14.88
207		54:5 (18:2/18:2/18:1)		898.8	601.5	30	15.19
208		54:4 (18:1/18:2/18:1)		900.8	601.4	30	15.83
209		54:3 (18:1/18:1/18:1)		902.8	603.5	30	16.45
210		54:2 (18:1/20:1/16:0)		904.8	605.5	30	17.09
211		54:1 (18:0/18:1/18:0)		906.8	605.3	30	17.76
212		56:8 (22:6/16:0/18:2)		920.7	575.5	30	14.65
213		56:7 (22:6/16:0/18:1)		922.8	577.3	30	15.34
214		56:6 (18:1/18:1/20:4)		924.8	625.5	30	15.68
215		56:5 (18:1/18:1/20:3)		926.8	627.3	30	16.22
216		56:4 (18:1/18:2/20:1)		928.8	629.5	30	16.61
217		56:3 (18:1/18:1/20:1)		930.8	631.5	30	17.2
218	AC	14:2	[M+H$^+$]	368.3	85.1	27	1.33
219		14:0		372.3	85.1	27	1.57
220		16:1		398.3	85.1	27	1.53
221		16:0		400.3	85.1	27	1.71
222		18:2		424.4	85.1	27	1.71
223		18:0		428.4	85.1	27	1.91

AC, d_7-acetylcarnitine; CE, cholesteryl ester; CER, ceramide; DAG, diacylglycerol; DG, diglycerides; Gal-SER, α-galactosylceramide; HSUL, hydroxysulfatide; Lac-CER, lactosylceramide; LPC, lysoPC; LPE, lysoPE; MG, monoglycerides; PC, phosphatidylcholine; PE, phosphatidylethanolamine; SM, sphingomyelin; SUL sulfatide, ; TAG, triacylglycerol; TG, triglycerides.
