# Supplementary figures and images for: Effect of Korean Red Ginseng on Plasma Ceramide Levels in Postmenopausal Women with Hypercholesterolemia: A Pilot Randomized Controlled Trial
Source: Metabolites. 2021 Jun 24;11(7):417. doi: 10.3390/metabo11070417 (PMC8307748; doi:10.3390/metabo11070417)

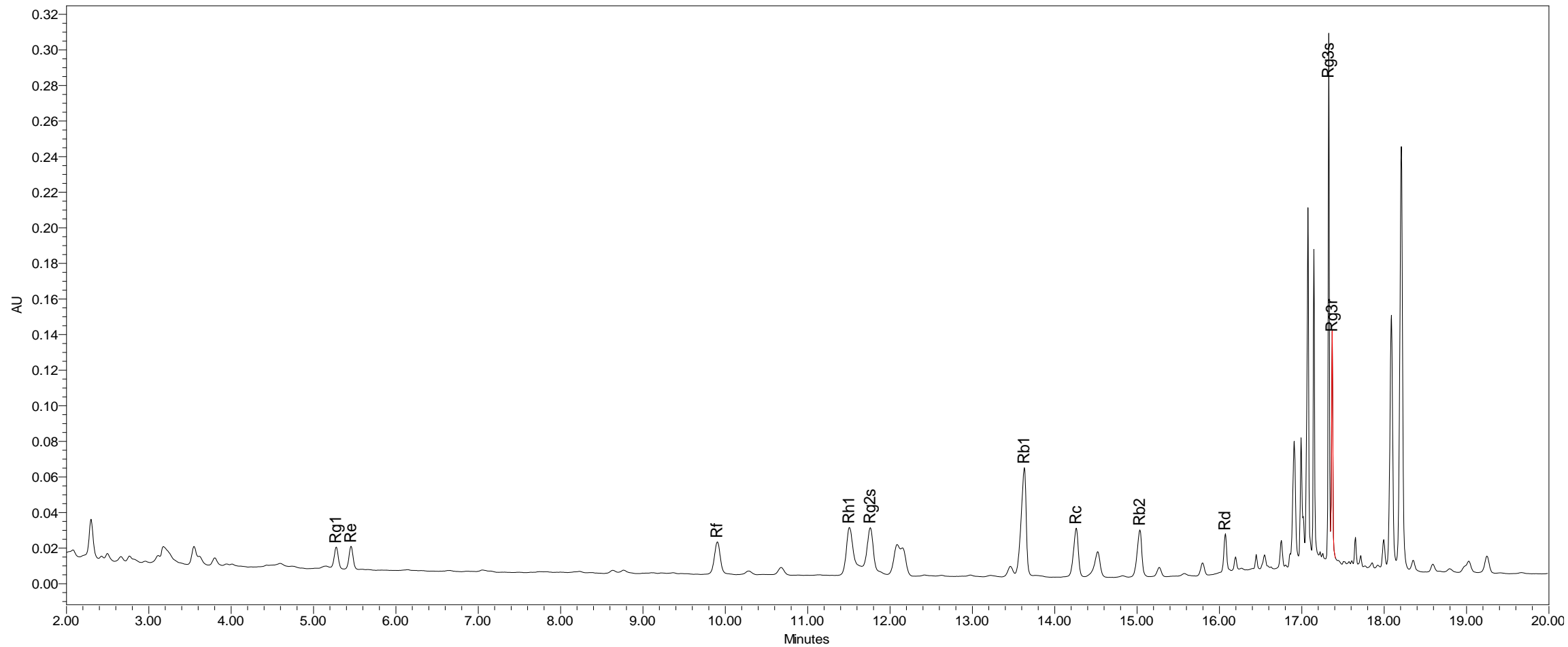

Supplement: Supplementary file 1 [file metabolites-11-00417-s001.zip › Figure S1.pdf]
